# Urinary collagen type I degradation products as common fibrosis biomarkers in chronic diseases

**DOI:** 10.1186/s10020-026-01648-5

**Published:** 2026-09-23

**Authors:** Ioanna K. Mina, Yaqza Hussain, Justyna Siwy, Lorenzo Catanese, Harald Rupprecht, Joachim Beige, Jan A. Staessen, Jochen Metzger, Frederik Persson, Peter Rossing, Christian Delles, Joost P. Schanstra, Ayman Bannaga, Antonia Vlahou, Harald Mischak, Ramesh P. Arasaradnam, Agnieszka Latosinska

**Affiliations:** 1 Mosaiques Diagnostics GmbH, Hannover, Germany; 2 Center of Systems Biology, Biomedical Research Foundation of the Academy of Athens, Athens, Greece; 3 University Hospitals Coventry & Warwickshire, Coventry, UK; 4 Department of Nephrology, Angiology and Rheumatology, Klinikum Bayreuth GmbH, Bayreuth, Germany; 5 Department of Nephrology, Medizincampus Oberfranken, Friedrich‐Alexander‐University Erlangen‐Nürnberg, Erlangen, Germany; 6 Kuratorium for Dialysis and Transplantation (KfH), Bayreuth, Germany; 7 Division of Nephrology and KfH Renal Unit, Hospital St Georg, Leipzig, Germany; 8 Martin-Luther University Halle-Wittenberg, Medical Clinic 2, Halle, Germany; 9 Non-Profit Research Institute Alliance for the Promotion of Preventive Medicine, Mechelen, Belgium; 10 Department of Cardiovascular Medicine, Shanghai Institute of Hypertension, Shanghai, China; 11 Key Laboratory of Hypertension, State Key Laboratory of Medical Genomics, National Research Centre for Translational Medicine, Ruijin Hospital, Shanghai, China; 12 Jiao Tong University School of Medicine, Shanghai, China; 13 Biomedical Research Group, Faculty of Medicine, University of Leuven, Leuven, Belgium; 14 Department of Clinical Medicine, University of Copenhagen, Copenhagen, Denmark; 15 Steno Diabetes Center Copenhagen, Herlev, Denmark; 16 School of Cardiovascular and Metabolic Health, University of Glasgow, Glasgow, UK; 17 Institute of Cardiovascular and Metabolic Disease, Institut National de La Santé Et de La Recherche Médicale (INSERM), Toulouse, France; 18 Université de Toulouse, Toulouse, France; 19 Queen Elizabeth Hospital Birmingham, Birmingham, UK; 20 Warwick Medical School, University of Warwick, Coventry, UK; 21 Leicester Cancer Research Centre, University of Leicester, Leicester, UK

**Keywords:** Fibrosis, Biomarkers, Urinary peptidomics, Collagen type I, Extracellular matrix remodelling, Capillary electrophoresis–mass spectrometry, Liver disease, Cirrhosis, Chronic kidney disease, Heart failure

## Abstract

**Background:**

Fibrosis, characterised by excessive accumulation of collagen type I (COL1), is a common feature of chronic diseases, including liver diseases (LDs), chronic kidney disease (CKD) and heart failure (HF). COL1 degradation products can be detected in urine by proteomics/ peptidomics analyses and may serve as non-invasive biomarkers of fibrosis. We aimed to identify a common molecular signature of fibrosis across these diseases that may ultimately guide interventions to slow disease progression and prevent organ damage.

**Methods:**

Using capillary electrophoresis coupled to mass spectrometry (CE-MS), naturally occurring COL1 degradation products (peptides) in the urine of patients with fibrotic disease, LDs (*n* = 127), CKD (*n* = 263) or HF (*n* = 187), were investigated and compared with the same number of matched controls. Disease-associated COL1 peptides were identified separately for each condition, and peptides showing consistent associations across the three diseases were selected to define a common fibrosis signature. A support vector machine model based on the selected peptides was developed and validated in independent cohorts of patients with LDs (*n* = 110), CKD (*n* = 93), HF (*n* = 32) and controls (*n* = 643).

**Results:**

We identified a common fibrotic signature consisting of 50 COL1 degradation products, mainly downregulated in fibrosis. A model based on these peptides achieved a strong performance, with an area under the receiver operating characteristic curve (AUC) of 0.935 (95% confidence interval (CI) 0.917–0.953, *p* < 0.0001) in an external validation cohort comprising pooled disease groups (LDs, CKD, and HF) and controls. Performance was maintained in LDs, CKD and HF, with AUCs of 0.917 (95% CI 0.890–0.944, *p* < 0.0001), 0.951 (95% CI 0.931–0.971, *p* < 0.0001) and 0.950 (95% CI 0.903–0.997, *p* < 0.0001), respectively. The model scores were significantly associated with fibrosis stage in LDs (*p* = 0.0097) and with interstitial fibrosis and tubular atrophy in CKD (*p* = 0.045).

**Conclusion:**

A model of urinary COL1 peptides captures a shared collagen degradation signature across organs and diseases, enabling the non-invasive assessment of fibrosis irrespective of its origin. As these peptides exclusively reflect collagen degradation, the findings suggest impaired collagen degradation as a driver in fibrosis. Future clinical studies are warranted to evaluate the utility of this model for early fibrosis detection and earlier implementation of anti-fibrotic interventions.

**Supplementary Information:**

The online version contains supplementary material available at https://doi.org/10.1186/s10020-026-01648-5.

## Background

Liver diseases (LDs), chronic kidney disease (CKD) and heart failure (HF) are major chronic diseases that frequently coexist, with dysfunction in one organ system contributing to disease progression in others, interactions that are increasingly recognised within the cardiovascular-kidney-metabolic syndrome (Lee et al. 2025). Fibrosis is an important component of the progression of these diseases and represents a common pathological process that also affects other organs, including the lung and skin (Wynn and Ramalingam 2012). Fibrosis is characterized by a dysregulated wound-healing response and excessive deposition of extracellular matrix components, particularly collagen type I (COL1) (Zhao et al. 2020), resulting from an imbalance between matrix production and degradation. Despite differences between organs and diseases, the central role of COL1 in fibrotic remodelling represents a shared biological basis that raises the possibility that therapeutic strategies targeting fibrotic processes in one organ system could be beneficial in other fibrotic diseases. Consequently, identifying a shared fibrosis molecular signature could provide a measure of ongoing fibrotic remodelling and facilitate earlier preventive and therapeutic interventions.

Specifically, previous studies have shown increased COL1 production in fibrotic liver (Thompson et al. 2011), kidney (Bülow and Boor 2019), and heart (Parichatikanond et al. 2020) tissues, primarily involving transforming growth factor-beta signalling (Parichatikanond et al. 2020; Antar et al. 2023; Kim et al. 2018). In parallel, COL1 degradation is mediated by specific proteases, including matrix metalloproteinases (MMPs) and cathepsins (McKleroy et al. 2013; Devos et al. 2023), which can be influenced either by changes in protease activity (for example, through regulation by tissue inhibitors of MMPs (Shan et al. 2023)) or by modifications to the COL1 itself that alter its susceptibility to proteolysis (Verzijl et al. 2000; Panwar et al. 2018).

COL1 consists of three chains, two collagen type I alpha 1 (COL1A1) chains and one collagen type I alpha 2 (COL1A2) chain, which are synthesised as procollagen chains consisting of an N-terminal propeptide, a central collagen domain and a C-terminal propeptide, each (Makareeva et al. 2014). Following their assembly into a triple helix and the cleavage of N- and C-propeptides, mature COL1 is formed (Makareeva et al. 2014). COL1 fragments are among the most abundant naturally occurring peptides in urine (Mavrogeorgis et al. 2021), with the majority (> 98%) being derived from mature COL1 rather than from the N- or C-terminal propeptides, indicating that they are products of collagen degradation rather than collagen synthesis. Many of these peptides have been associated with LDs (Bannaga et al. 2020), CKD (Mavrogeorgis et al. 2022; Good et al. 2010; Mina et al. 2025) and HF (He et al. 2021), forming a basis for peptide-based biomarker approaches. Indeed, models based on urinary peptides have already been developed for the detection of organ specific fibrosis in liver (Bannaga et al. 2020) and kidney (Catanese et al. 2021), demonstrating their applicability as non-invasive biomarkers.

Fibrosis is responsible for up to 45% of deaths in the developed countries (Ku et al. 2024), and globally approximately 1 in 4 people have liver fibrosis, 1 in 6 have kidney fibrosis, and 1 in 60 have heart fibrosis (Zhao et al. 2020). Despite this high prevalence, biopsy remains the gold standard for diagnosing fibrosis in these organs (Canivet and Boursier 2023; Abdelhameed et al. 2024; Wan et al. 2024; Zhu et al. 2022). However, it is an invasive procedure that carries risks of complications, including, in rare cases, death (Canivet and Boursier 2023; Wan et al. 2024). Additionally, biopsies cannot be repeated multiple times (for the purpose of monitoring) and are prone to sampling errors (Wan et al. 2024; Zhu et al. 2022).

Collectively, we hypothesised that: 1) fibrosis may be the consequence of attenuation of COL1 degradation, and 2) a generic fibrosis signature reflecting the collagen degradation process exists in the urinary peptidome, independent of the affected organ. To test these hypotheses, we investigated the association between naturally occurring COL1 degradation products in urine and fibrotic-related chronic diseases, with the aim of developing a non-invasive, urine-based method to detect an ongoing fibrotic remodelling across organs, rather than replace organ-specific diagnostics, and ultimately support earlier treatment decisions before irreversible organ damage occurs.

## Methods

### Capillary electrophoresis coupled to mass spectrometry (CE-MS) data

Proteome analysis was performed as described previously (Mischak et al. 2013). In brief, a P/ACE MDQ CE coupled to a micro-TOF–MS was used for CE-MS analysis. The raw CE-MS data were evaluated using the MosaFinder software assigning the detected signal in silico to the list of 21,559 peptides, with > 5000 having information about the amino acid sequence (Latosinska et al. 2019). Twenty-nine collagen fragments generally unaffected in disease were used as internal standards for normalizing peptide intensities (Jantos-Siwy et al. 2009).

### Study population

Anonymized urinary peptidome, and phenotypic data were extracted from the Human Urinary Proteome Database (Latosinska et al. 2019). Due to the known associations of age (Martens et al. 2021), sex (Mina et al. 2024), diabetes (Rossing, et al., 2008; Tofte et al. 2020) and kidney function (Mavrogeorgis et al. 2022) with the urinary peptides, cases were matched based on these characteristics with their respective controls. For biomarker discovery, patients with different types of LDs (Table 1) and confirmed liver fibrosis established by a combination of liver ultrasound, fibroscan and laboratory markers (e.g. aspartate aminotransferase to platelet ratio index), as well as liver histology in cases of uncertainty (study by (Bannaga et al. 2020)) were matched in a ratio 1 to 1 with control individuals from the general population studies (FLEMENGHO (Zhang et al. 2014) and Generation Scotland (Smith et al. 2013)) based on age and sex. Because all patients with LDs had an estimated glomerular filtration rate (eGFR) above 60 mL/min/1.73 m^2^, only control individuals with eGFR > 60 mL/min/1.73 m^2^ were selected. Similarly, CKD patients with different aetiologies (Table 1) and interstitial fibrosis and tubular atrophy (IFTA) ≥ 15% (biopsy-based histological assessment) were extracted from the study by Catanese et al. (Catanese et al. 2021). Because many of these patients also had type II diabetes, they were matched with individuals from the general population study (FLEMENGHO (Zhang et al. 2014)) and from the type II diabetes study (PRIORITY (Tofte et al. 2020)) based on age, sex and the presence of diabetes. Matching for eGFR could not be performed due to the disease-specific distribution of kidney function in the CKD cohort. Although no data from patients with confirmed heart fibrosis were available, previous studies have established the presence of heart fibrosis in the majority of patients with HF with preserved ejection fraction (HFpEF) (Mohammed et al. 2015; Hahn et al. 2020). Therefore, patients with HFpEF and control individuals matched for multiple parameters, including age, sex, eGFR and the presence of diabetes, were extracted from the study by He et al. (He et al. 2021).

**Table 1:** Cohort characteristics. Values are presented as median [interquartile range] for numerical variables or n (%) for categorical variables. Only characteristics with a prevalence greater than 70% in any given cohort are reported. Comparisons were made between cases and their corresponding control groups using the Mann–Whitney test for continuous variables and the Chi‐squared test for categorical variables

	DISCOVERY						VALIDATION			
	**LIVER CASES**	**LIVER CONTROLS**	**KIDNEY CASES**	**KIDNEY CONTROLS**	**HEART CASES**	**HEART CONTROLS**	**LIVER CASES**	**KIDNEY CASES**	**HEART CASES**	**CONTROLS**
	***n* = 127**	***n* = 127**	***n* = 263**	***n* = 263**	***n* = 187**	***n* = 187**	***n* = 110**	***n* = 93**	***n* = 32**	***n* = 643**
Main clinical characteristics										
Male sex	83 (65.4%)	80 (63.0%)	167 (63.5%)	160 (60.8%)	101 (54.0%)	103 (55.1%)	63 (57.3%)	68 (73.1%)^a^	12 (37.5%)	350 (54.4%)
Age (years)	59.0 [47.0–67.0]	59.7 [50.1–69.0]	63.3 [51.3–72.7]	58.2 [52.9–68.8]	72.7 [68.3–78.2]	73.0 [68.0–76.5]	61.0 [54.2–69.0]^a^	64.0 [46.4–73.8]^a^	81.7 [78.2–86.6]^a^	50.6 [39.5–61.6]
Diabetes	0 (0.0%)	0 (0.0%)	52 (19.8%)	53 (20.2%)	77 (41.2%)	72 (38.5%)	26 (23.6%)^a^	15 (16.1%)^a^	7 (21.9%)^a^	28 (4.4%)
Hypertension	-	36 (28.3%)	118 (45.2%)^b^	122 (46.4%)	113 (60.4%)	118 (63.1%)	23 (20.9%)^a^	43 (46.2%)	17 (53.1%)	256 (39.8%)
CAD	-	-	-	-	78 (41.7%)^a^	23 (15.0%)^b^	3 (2.7%)	-	14 (43.8%)	-
eGFR (mL/min/1.73 m^2^)	-	83.0 [73.1–91.4]	19.8 [11.0–36.2]^a^	78.0 [71.0–89.7]	61.8 [48.0–77.5]	65.7 [46.4–80.2]	-	17.8 [10.7–31.3]^a^	58.8 [40.7–66.3]^a^	87.7 [78.2–96.4]
BMI	-	26.9 [24.4–30.1]^b^	-	27.4 [25.0–29.9]^b^	31.0 [27.1–35.8]^a,b^	26.6 [23.9–30.4]^b^	30.3 [26.7–34.7]^a,b^	-	27.0 [24.1–30.5]^a^	25.5 [23.2–28.4]
LD aetiology										
NAFLD	19 (15.0%)	-	-	-	-	-	18 (16.4%)	-	-	-
NASH	8 (6.3%)	-	-	-	-	-	-	-	-	-
CIRR	61 (48.0%)	-	-	-	-	-	92 (83.6%)	-	-	-
CIRR & Cancer	39 (30.6%)	-	-	-	-	-	-	-	-	-
Liver fibrosis stage										
F1	-	-	-	-	-	-	10 (9.1%)	-	-	-
F2	-	-	-	-	-	-	3 (2.7%)	-	-	-
F3	-	-	-	-	-	-	3 (2.7%)	-	-	-
F4	100 (78.6%)	-	-	-	-	-	94 (85.5%)	-	-	-
CKD aetiology										
HINP	-	-	68 (25.9%)	-	-	-	-	20 (21.5%)	-	-
IGANP	-	-	48 (18.3%)	-	-	-	-	33 (35.5%)	-	-
DNPNOD	-	-	19 (7.2%)	-	-	-	-	7 (7.5%)	-	-
INTN	-	-	19 (7.2%)	-	-	-	-	4 (4.3%)	-	-
EPGN	-	-	16 (6.1%)	-	-	-	-	4 (4.3%)	-	-
Other aetiology	-	-	93 (35.4%)	-	-	-	-	25 (26.9%)	-	-
IFTA%	-	-	30.0 [20.0–45.0]	-	-	-	-	30.0 [22.5–50.0]	-	-
NYHA class										
NYHA0	-	-	-	-	0 (0.0%)^a^	115 (86.5%)^b^	-	-	0 (0.0%)	-
NYHA1	-	-	-	-	41 (21.9%)	18 (13.5%)^b^	-	-	3 (9.4%)	-
NYHA2	-	-	-	-	105 (56.1%)^a^	0 (0.0%)^b^	-	-	19 (59.4%)	-
NYHA3	-	-	-	-	37 (19.8%)^a^	0 (0.0%)^b^	-	-	9 (28.1%)	-
NYHA4	-	-	-	-	4 (2.1%)	0 (0.0%)^b^	-	-	1 (3.1%)	-
Additional markers										
ALT (U/L)	37.0 [27.0–60.0]^b^	-	-	-	-	-	30.0 [20.8–46.0]^b^	-	-	-
AST (U/L)	44.0 [31.0–65.5]^b^	-	-	-	-	-	-	-	-	-
ALP (U/L)	113.0 [84.0–182.0]^b^	-	-	-	-	-	100.0 [74.0–145.0]^b^	-	-	-
GGT (U/L)	118.5 [63.0–205.2]^b^	-	-	-	-	-	-	-	-	-
AFP (μg/L)	3.0 [2.0–8.0]^b^	-	-	-	-	-	-	-	-	-
INR	1.1 [1.0–1.3]^b^	-	-	-	-	-	1.0 [1.0–1.1]^b^	-	-	-
Albumin (g/L)	39.0 [34.0–45.0]^b^	-	-	-	-	-	43.0 [38.0–46.0]^b^	-	-	-
DBP (mmHg)	-	81.0 [74.0–87.6]	-	81.6 [75.2–86.8]^b^	70.0 [64.0–82.0]	75.0 [68.0–80.0]	-	-	57.0 [53.8–59.5]^a^	79.2 [73.0–86.0]
SBP (mmHg)	-	134.0 [120.6–147.3]	-	132.0 [122.0–142.8]^b^	135.0 [121.5–154.0]	139.0 [127.5–151.5]	-	-	128.0 [118.0–140.0]	128.0 [117.6–140.8]
Proteinuria (mg/24 h)	-	-	2400.0 [716.1–5283.0]^b^	-	-	-	-	-	-	-
Albuminuria (mg/g creatinine)	-	-	-	-	-	-	-	1671.5 [520.5–3494.2]^b^	-	-
Ejection fraction	-	-	-	-	56.0 [53.0–62.0]	-	-	-	58.0 [54.0–62.2]	-
NT‐proBNP (pg/mL)	-	-	-	-	692.0 [335.0–1155.5]	-	-	-	1103.0 [647.8–2536.5]	-
BNP (pg/mL)	-	-	-	-	336.0 [154.5–522.8]	-	-	-	390.5 [211.2–573.2]	-
Heart rate (bpm)	-	-	-	61.6 [54.6–68.5]^b^	70.0 [60.0–80.0]	-	-	-	66.0 [55.0–76.5]	-

^a^indicates significant differences (p < 0.05) between cases and their respective controls

^b^indicates that the variable was not available for 100% of individuals in the cohort

*Abbreviations*: *ALT* alanine aminotransferase, *AFP* alpha-fetoprotein, *ALP* alkaline phosphatase, *AST* aspartate aminotransferase, *BMI* body mass index, *BNP* B-type natriuretic peptide, *CAD* coronary artery disease, *CIRR* cirrhosis, *CKD* chronic kidney disease, *DBP* diastolic blood pressure, *DNPNOD* nodular diabetic nephropathy, *eGFR* estimated glomerular filtration rate, *EPGN* endocapillary proliferative glomerulonephritis, *GGT* gamma-glutamyl transferase, *HINP* hypertensive ischemic nephropathy, *IGANP* IgA nephropathy, *IFTA* interstitial fibrosis and tubular atrophy, *INR* international normalized ratio, *INTN* interstitial nephritis, *LD* liver disease, *NAFLD* non-alcoholic fatty liver disease, *NASH* non-alcoholic steatohepatitis, *NT-proBNP* N-terminal pro-B-type natriuretic peptide, *NYHA* New York Heart Association, *SBP* systolic blood pressure

For the model validation independent datasets from patients with confirmed liver fibrosis (stages F1-F4), patients with confirmed IFTA ≥ 15% and patients with HFpEF were extracted from the TENDENCY (Hussain et al. 2023) study, study by Frantzi et al. (Frantzi et al. 2026) and study by He et al. (He et al. 2021), respectively (Table 1). Liver and kidney fibrosis were assessed using the same methods applied for the discovery cohorts. For this independent validation, control individuals, who were not included in the biomarker discovery phase, without clinical signs of organ damage and with an eGFR above 60 mL/min/1.73 m^2^, were extracted from the general population studies FLEMENGHO (Zhang et al. 2014) and Generation Scotland (Smith et al. 2013).

Fibrosis could not be excluded in the control group (either in discovery or validation), as invasive or dedicated fibrosis assessments are not routinely performed in these populations. Only datasets from individuals above 18 years old were investigated. Cases and controls were matched using the MatchIt package in R (Ho et al. 2011).

### Statistical analysis and model generation

Statistical analysis was performed comparing COL1 peptides (*i.e.* COL1A1, COL1A2) between cases and controls within each disease group. In each comparison, only peptides with a frequency ≥ 30% in cases or controls were considered. The frequency threshold of 30% has been found optimal in previous peptidome studies to address missing values while maintaining high coverage (An et al. 2023). Disease-associated peptides were identified using the non-parametric Mann–Whitney test and resulting p-values were adjusted using the Benjamini–Hochberg method (Benjamini and Hochberg 1995). Statistical tests were conducted under two conditions: 1) without replacing the missing values and 2) after replacing missing values with zero, and only changes with a significant adjusted p-value in the analysis after the replacement of missing values with zero, which agreed in fold change direction in the analysis without replacement of missing values, were retained. The results of the disease-specific analyses were then used for the selection of common COL1 fibrosis biomarkers. For ranking common fibrosis biomarkers based on significance, raw p-values across the three organ comparisons were combined using Fisher’s method. The analyses were conducted using the base R functions wilcox.test and p.adjust and the poolr package.

**Fig. 1:**
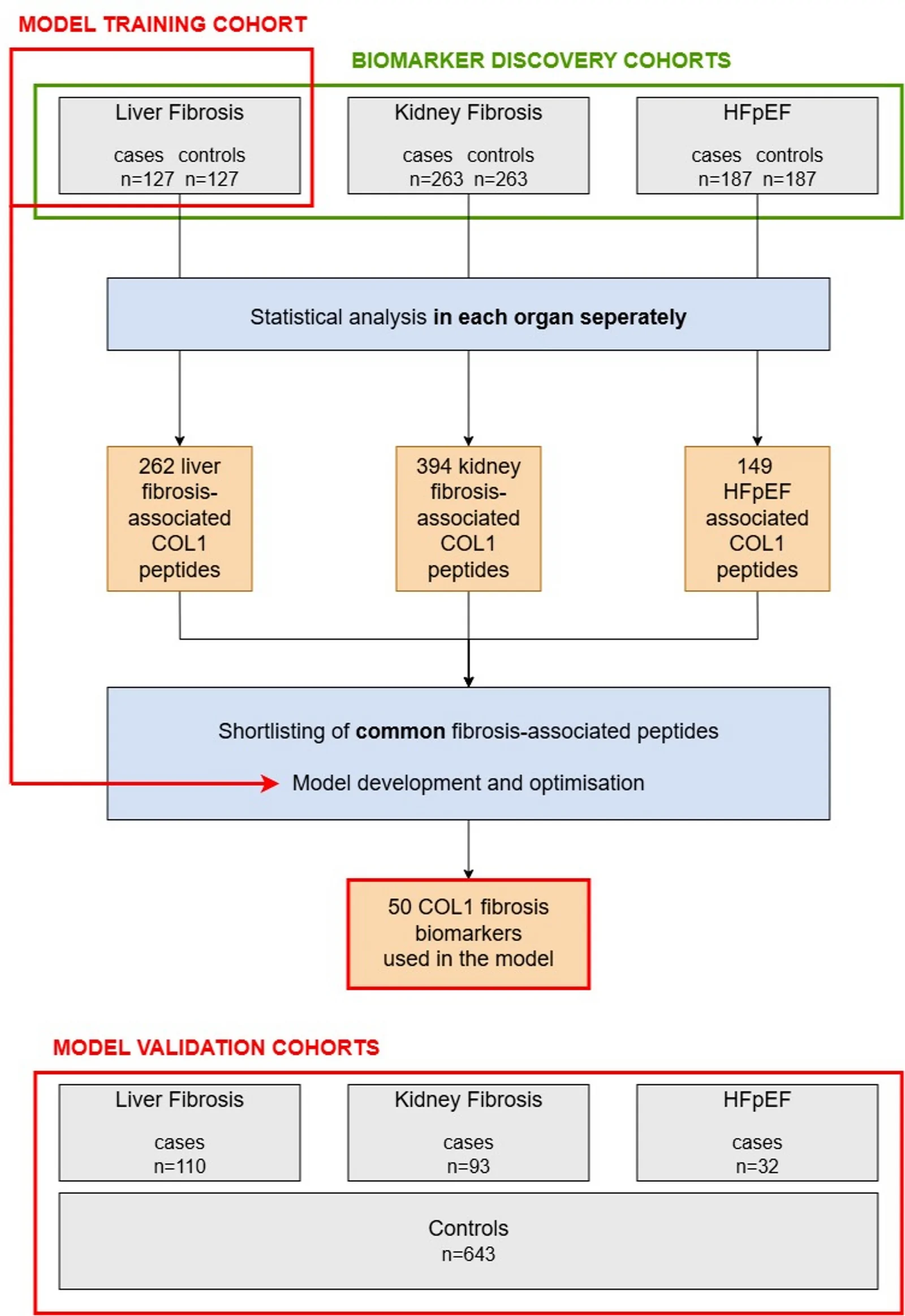
Study design. To identify fibrosis-associated COL1 peptides, separate case–control analyses were performed for liver fibrosis, kidney fibrosis, and heart failure with preserved ejection fraction (HFpEF). Peptides detected in ≥ 30% of samples in cases and/or controls were selected for each organ. Case–control comparisons were conducted using two approaches: without imputation of missing values and with missing values replaced by zero. Peptides were retained as organ-specific biomarkers if they showed a significant adjusted p-value in the analysis with imputation and a concordant fold change (FC) direction in the analysis without imputation. Peptides meeting the 30% frequency threshold across all three organs were considered candidate common fibrosis biomarkers and were further selected based on (i) statistical significance in at least two organ comparisons and (ii) consistent FC direction across all three. The top 60 peptides, ranked by Fisher’s combined p-values across organs, were further reduced to a final 50-peptide model using take-one-out cross-validation in the liver fibrosis cohort for biomarker discovery. A support vector machine (SVM) model was then trained based on these 50 fibrosis-associated peptides in the liver fibrosis cohort used for biomarker discovery. The model was validated in independent liver fibrosis, kidney fibrosis, and HFpEF cohorts. Abbreviations: COL1: collagen type I, HFpEF: heart failure with preserved ejection fraction

Based on selected biomarkers, a model using a support vector machine (SVM) algorithm integrated into the MosaCluster software (Mischak et al. 2013) was developed and optimised using take-one-out cross-validation. The model was trained using the same liver fibrosis patients and their respective controls that were used during biomarker development. The liver fibrosis cohort was selected for model training because fibrosis in this cohort was confirmed and eGFR was above 60 mL/min/1.73 m^2^, reducing the potential influence of impaired kidney function on urinary peptide levels. To select the SVM hyperparameters, we screened a range of values for C (20–20,000) and gamma (0.000002–0.002). The optimal parameter combination (C: 2560; gamma 0.000002^)^ was selected based on overall cross-validation performance, with preference given to models with higher C, lower gamma, and fewer number of support vectors. The optimal model score cut-off was determined based on the cross-validation results using the Youden index. The model was then validated in independent datasets of patients with liver fibrosis, kidney fibrosis or HFpEF and individuals from the general population (Fig. 1). The scoring of the validation cohorts based on the model was conducted in the MosaDiagnostics software (Mischak et al. 2013). The R package pROC was used to determine and plot the area under the receiver operating characteristic curve (AUC), including its confidence interval (CI), for the generated model. To assess the statistical significance of the AUC, the roc.area function from the verification package was used. Confidence intervals for sensitivity and specificity were calculated using the exact Clopper–Pearson method (https://epitools.ausvet.com.au/ciproportion).

Differences in model scores among the chronic disease and general population control cohorts were assessed using the Kruskal–Wallis test followed by Conover post hoc comparisons. The association between model scores and liver fibrosis stage was assessed using the Mann–Whitney test, while the correlation of the model scores with IFTA% was evaluated using Spearman’s correlation. These analyses were conducted using the kruskal.test, kwAllPairsConoverTest, wilcox.test and cor.test functions in R.

Data visualizations, including plots and heatmaps, were generated using the ggplot2 and gplots packages. R version 4.3.3 was used for all analyses and visualisations.

## Results

### Cohort characteristics

The case cohorts used for biomarker discovery are based on previously published studies, and were comprised of 127 patients with LDs (mainly cirrhosis), 263 patients with CKD (different aetiologies) and 187 patients with HF (HFpEF), along with the same number of matched control individuals. Detailed characteristics of these subjects are presented in Table 1. Of the 127 patients with LDs, 100 (78.6%) had fibrosis stage 4. The CKD cohort had a median IFTA of 30%, with an interquartile range (IQR) of 20–45%. Among the 187 datasets from patients with HF, 41 (21.9%) were classified as New York Heart Association (NYHA) class 1, 105 (56.1%) as NYHA class 2, 37 (19.8%) as NYHA class 3, and 4 (2.1%) as NYHA class 4. The same liver fibrosis cases with their matched controls were also used for the model training (Fig. 1).

The model validation cohort was comprised of remaining subjects that could not be matched with controls during discovery and additional datasets generated after the publications of the initial studies. It included in total 235 cases and 643 controls. The cases were comprised of 110 patients with LDs (mainly cirrhosis, fibrosis stage 4), 93 patients with CKD (different aetiologies, median IFTA of 30%, [IQR 22.5–50%]) and 32 patients with HF (HFpEF, mainly NYHA stage 2 and 3). Detailed characteristics of the subjects are presented in Table 1.

### Liver fibrosis, kidney fibrosis and HFpEF share a common COL1 degradation signature

To date, 1097 naturally occurring COL1-derived peptides have been identified in urine, with 788 of them originating from COL1A1 and 309 from COL1A2. The 98.7% of these peptides are derived from the mature COL1A1 and COL1A2 chains, consequently reflecting the degradation of COL1. The abundance of these peptides was assessed in multiple cohorts, as shown in the study design in Fig. 1.

**Fig. 2:**
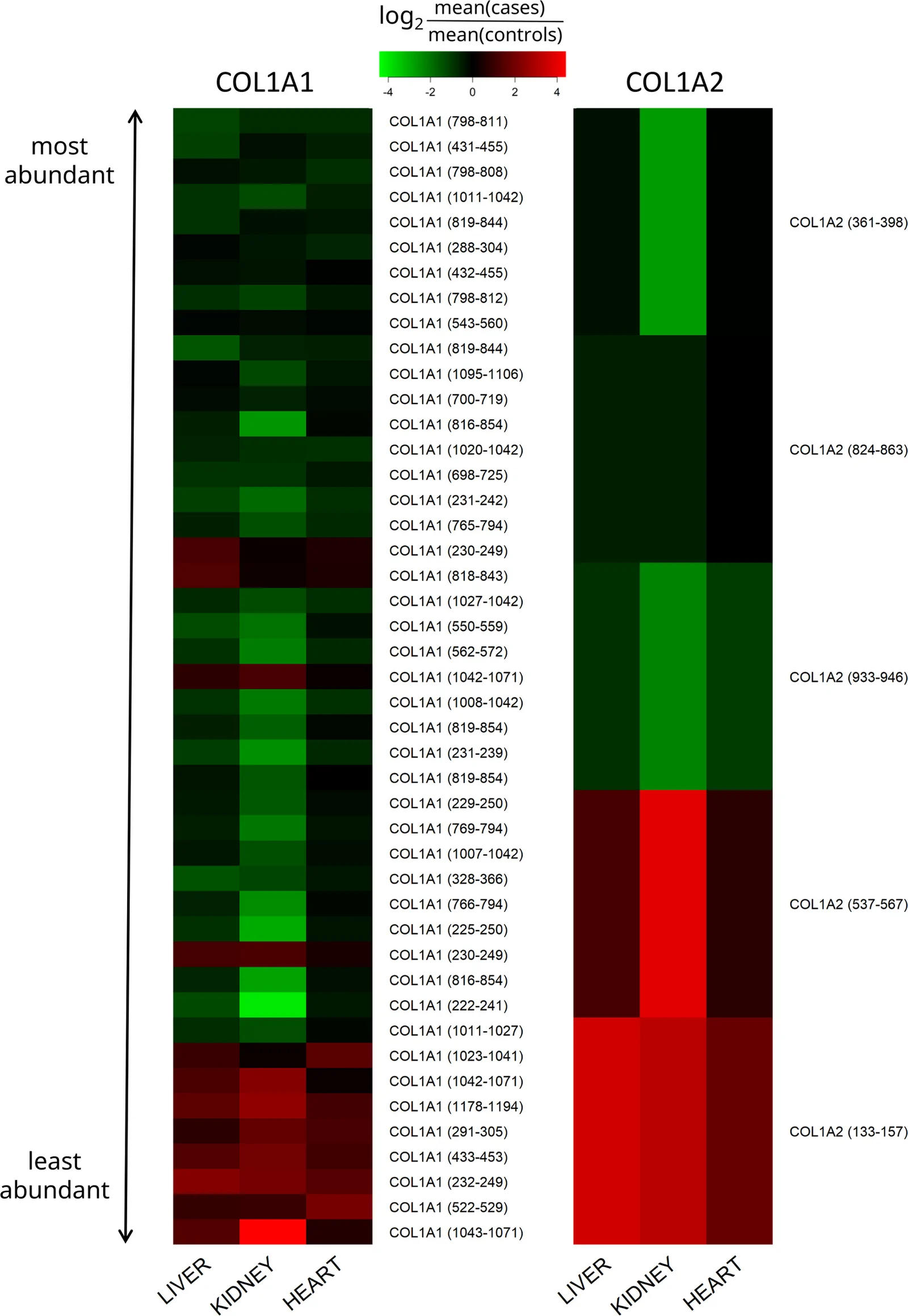
Regulation of urinary peptides in fibrosis. Heatmaps depicting the log_2_ fold change of the 50 fibrosis-associated urinary peptides across liver fibrosis, kidney fibrosis and heart failure with preserved ejection fraction (HFpEF) biomarker discovery cohorts. More than one peptide may share the same start and stop amino acid but differ in mass due to hydroxylation of proline residues. The exact sequences of the fibrosis-associated peptides, including residue modifications are provided in Additional File 1; Supplementary Table 4. Peptides are ordered based on the median of their mean abundances in controls across the three organ comparisons, with the most abundant peptides displayed at the top of the heatmaps. Abbreviations: COL1A1: collagen type I alpha 1 chain, COL1A2: collagen type I alpha 2 chain

Statistical analysis of cases and matched controls in the discovery cohorts identified 262 COL1 peptides associated with liver fibrosis (Additional File 1; Supplementary Table 1), 394 associated with kidney fibrosis (Additional File 1; Supplementary Table 2) and 149 associated with HFpEF (Additional File 1; Supplementary Table 3). Since many of these peptides were associated with more than one disease (Additional File 2; Supplementary Fig. 1) the analysis resulted in 526 unique peptides, of which 403 were detected with a frequency greater than 30% (in either cases or controls) across all comparisons. Given differences in cohort sizes, and consequently statistical power, additional selection criteria were applied to ensure robustness. A subset of 90 peptides was identified based on the following conditions: (i) statistical significance in at least two comparisons and (ii) consistent fold change direction across all three comparisons. From this subset, the 60 peptides with the lowest Fisher’s combined p-values across organ comparisons were retained and subsequently reduced to a final set of 50 peptides using a take-one-out cross-validation approach (Fig. 2, Additional File 1; Supplementary Table 4). The majority (*n* = 45) peptides were derived from the COL1A1 chain, of which 33 were downregulated and 12 upregulated in fibrosis. The remaining 5 peptides were derived from COL1A2, with 3 being downregulated and 2 being upregulated in fibrosis (Fig. 2, Additional File 1; Supplementary Table 4). All of the 50 fibrosis-associated peptides were fragments of the mature COL1A1 and COL1A2 chains, thus reflecting COL1 degradation.

### A model based on the common COL1 degradation signature is able to detect fibrosis, across different organs and diseases

**Fig. 3:**
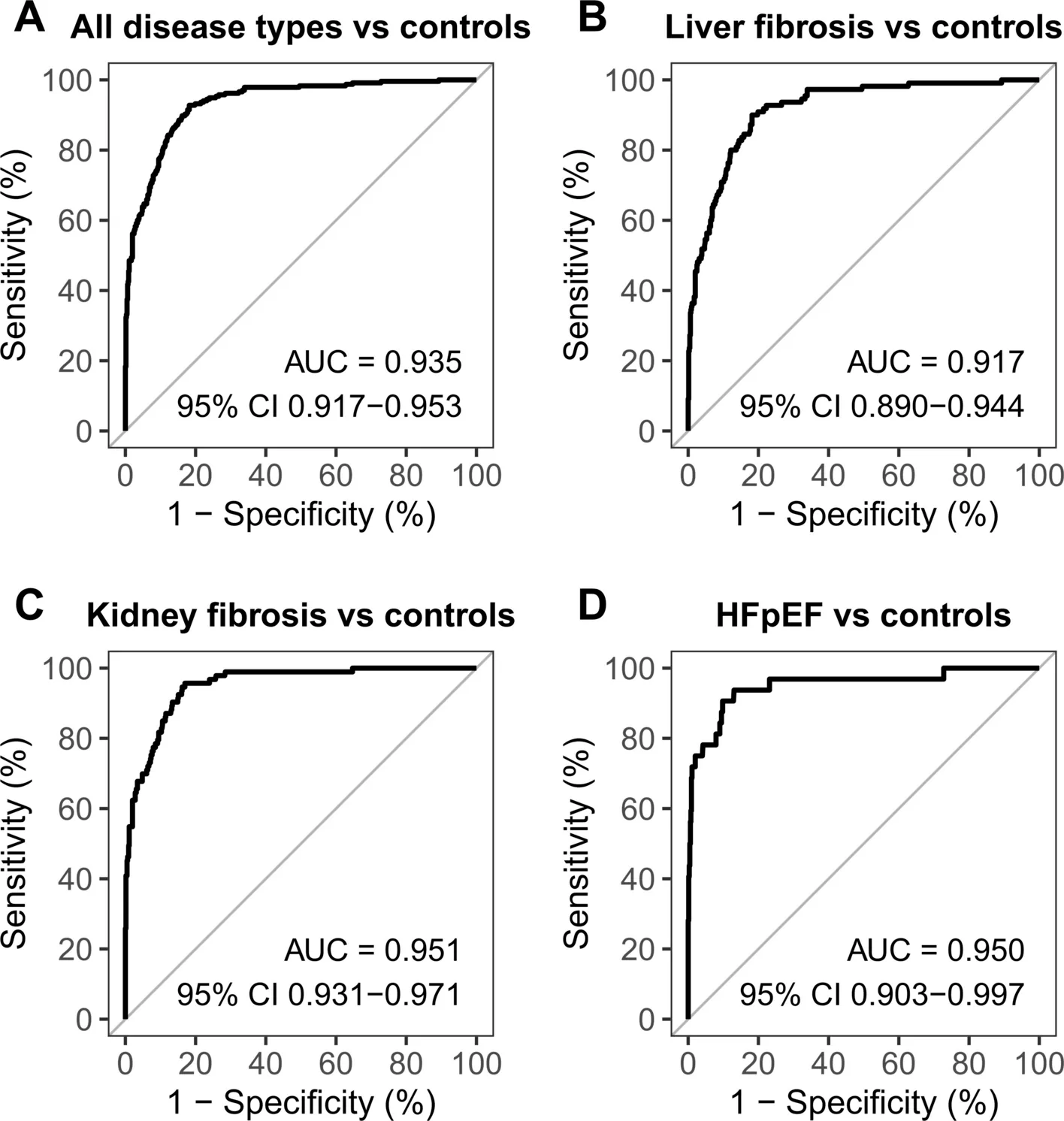
Performance of the collagen type I-based fibrosis model. **A** Receiver operating characteristic (ROC) curve showing model performance in the full validation cohort comprising patients with liver fibrosis, kidney fibrosis and HFpEF (*n* = 235) and individuals from the general population (*n* = 643). (B-D) ROC curves for organ-specific validation subgroups: liver fibrosis (*n* = 110), kidney fibrosis (*n* = 93), and HFpEF (*n* = 32), each evaluated against a shared control group (*n* = 643). Abbreviations: AUC: area under the receiver operating characteristic curve, CI: confidence interval, HFpEF: heart failure with preserved ejection fraction

An SVM model was generated using the final set of 50 COL1 degradation biomarkers identified through take-one-out cross-validation, with the liver fibrosis discovery cohort used as the training set (Additional File 2; Supplementary Fig. 2). The model demonstrated good discriminatory performance, achieving an AUC of 0.935 (95% CI 0.917–0.953, *p* < 0.0001) in the validation cohort for discriminating patients with all three chronic diseases from controls (Fig. 3A). Importantly, the model maintained consistently high performance when evaluated separately in patients with liver fibrosis (*n* = 110), kidney fibrosis (*n* = 93), and HFpEF (*n* = 32), with corresponding AUCs of 0.917 (95% CI 0.890–0.944, *p* < 0.0001), 0.951 (95% CI 0.931–0.971, *p* < 0.0001), and 0.950 (95% CI 0.903–0.997, *p* < 0.0001), respectively, each compared against a shared control group (*n* = 643) (Fig. 3B-D). Comparison of these AUCs showed that discriminatory performance was significantly higher for kidney than liver fibrosis (*p* = 0.026), while the differences between liver fibrosis and HFpEF and between kidney fibrosis and HFpEF were not significant (Supplementary Table 5).

To assess whether the higher number of available controls compared to cases in the validation cohort influenced model performance, the analysis was repeated 100 times using balanced case–control datasets generated by random sampling, both for all disease groups combined and for each disease group separately. The mean AUCs were closely corresponding to those obtained using the full validation cohort (Additional File 1; Supplementary Table 6).

### Model scores based on the common COL1 degradation signature reflect fibrosis severity across organs

**Fig. 4:**
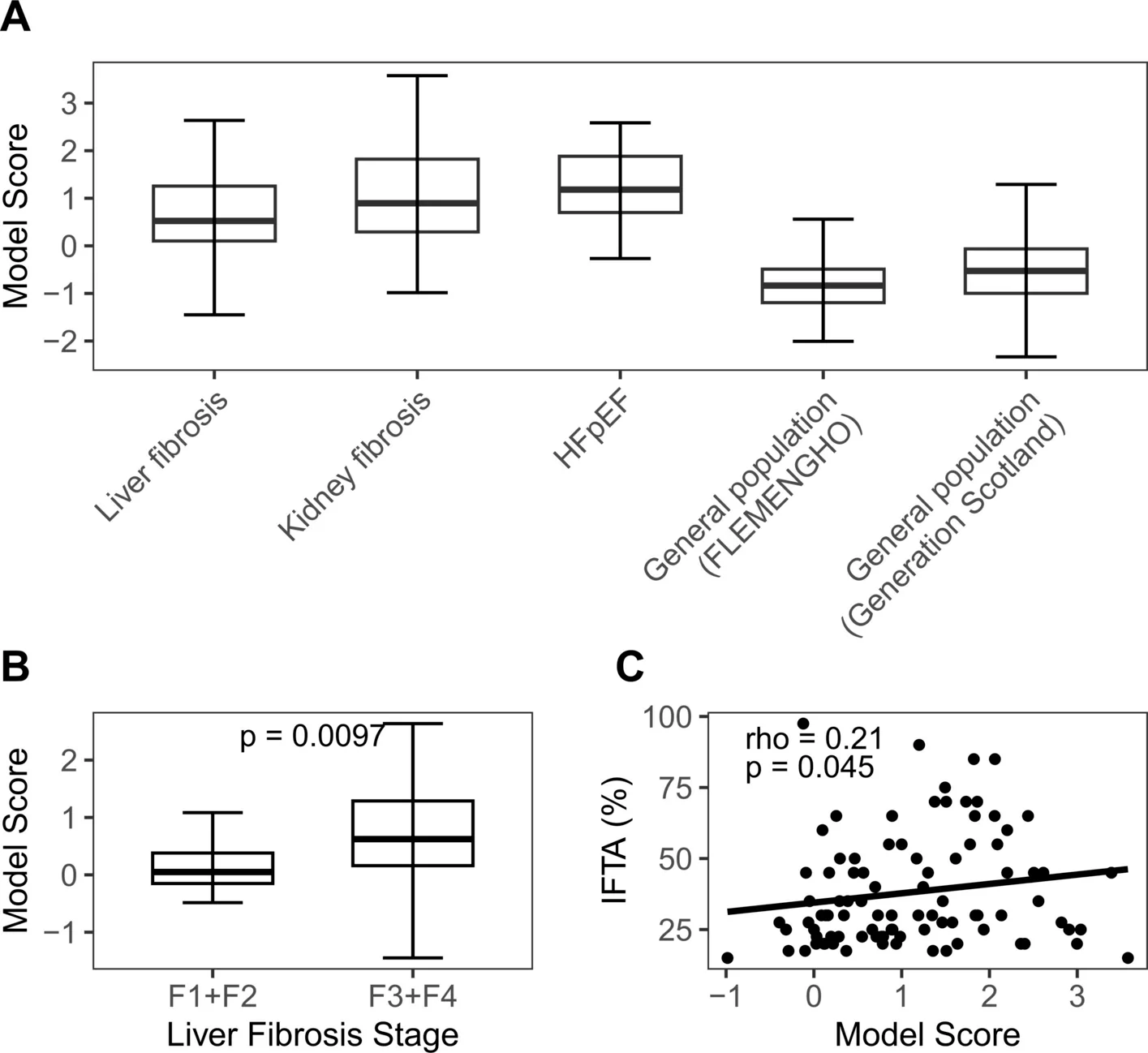
Distribution and clinical relevance of collagen type I-based fibrosis model scores. **A** Boxplots showing model scores across the validation cohorts: liver fibrosis (*n* = 110), kidney fibrosis (*n* = 93), heart failure with preserved ejection fraction (HFpEF, *n* = 32), and two general population cohorts: FLEMENGHO (*n* = 399) and Generation Scotland (*n* = 244). Differences among the five groups were assessed using the Kruskal–Wallis test followed by Conover post hoc test. Scores did not differ significantly among the three disease groups (all Conover *p* > 0.05), while each fibrosis cohort differed significantly from both general population control cohorts (all Conover *p* < 0.0001). Complete comparison results are provided in Additional File 1; Supplementary Table 7. **B** Boxplots of model scores stratified by fibrosis stage in the liver fibrosis validation cohort, comparing patients with stage F1-F2 (*n* = 13) and stage F3–F4 (*n* = 97) fibrosis. Group differences were evaluated using the Mann–Whitney test. **C** Scatter plot showing the correlation between model scores and the percentage of interstitial fibrosis and tubular atrophy (IFTA) in the kidney fibrosis validation cohort (*n* = 93). Correlation was assessed using the Spearman’s test. Abbreviations: HFpEF: heart failure with preserved ejection fraction, IFTA: interstitial fibrosis and tubular atrophy, rho: Spearman’s correlation coefficient

Consistent with the observed discriminatory performance, the distribution of model scores across fibrosis cohorts and general population controls is presented in Fig. 4A. Model scores did not differ significantly among the liver fibrosis, kidney fibrosis, and HFpEF cohorts (Conover post hoc test, all *p* > 0.05). In contrast, each disease cohort differed significantly from both general population control cohorts (all *p* < 0.0001). Complete comparison results are provided in Additional File 1; Supplementary Table 7.

Furthermore, the model scores demonstrated a significant association with the liver and kidney fibrosis stage. Specifically, patients with advanced liver fibrosis (stage 3 or 4; *n* = 97) had higher median scores of 0.62 [IQR 0.16—1.29], compared to those with early-stage fibrosis (stage 1 or 2; *n* = 13), who had lower median scores of 0.05 [IQR −0.15—0.38] (*p* = 0.0097; Mann–Whitney test; Fig. 4B). Consistent with these findings, the model scores showed a significant positive correlation with the percentage of IFTA in the kidney fibrosis validation cohort (*n* = 93; Spearman’s rho = 0.21, *p* = 0.045; Fig. 4C).

## Discussion

Although increased COL1 synthesis is widely recognised as a major contributor to fibrosis (Thompson et al. 2011; Bülow and Boor 2019; Parichatikanond et al. 2020), the role of impaired collagen degradation has received much less attention, particularly when considering similarities across different chronic diseases. Analysis of the urinary peptidome provides a way to explore this aspect, as a large proportion of naturally occurring urinary peptides originating from COL1 is derived from the mature collagen rather than from the N- or C-terminal propeptides released during collagen synthesis (Mavrogeorgis et al. 2022), indicating that they are products of COL1 degradation. Associations between urinary COL1 peptides and fibrotic disease in the liver (Bannaga et al. 2020), kidney (Mavrogeorgis et al. 2022; Good et al. 2010; Mina et al. 2025) and heart (He et al. 2021) have been reported previously, supporting their relevance in this context. On this basis, COL1-derived peptides have been also used to develop models for the non-invasive assessment of fibrosis in the liver (Bannaga et al. 2020) and kidney (Catanese et al. 2021) (comparison with the common fibrosis model in Additional File 1; Supplementary Table 8). Moreover, our previous study (Mina et al. 2025), focusing on COL1A1-derived (*i.e.* the specific COL1A1 chain) urinary peptides, showed that early stage fragments in the collagen degradation process, likely directly derived from the COL1A1 molecule as a result of endopeptidase activity, were consistently downregulated in patients with CKD, suggesting that the accumulation of COL1 in fibrosis is partly the result of an attenuated COL1 degradation process (Mina et al. 2025). However, a small proportion of peptides from later degradation steps, likely derived from larger peptides through exopeptidase activity, were upregulated (Mina et al. 2025), which might explain why the majority of the 50 common COL1-derived biomarkers identified in the present study were downregulated in fibrosis, while a smaller proportion were upregulated. A study using a unilateral ureteral obstruction mouse model further supported the hypothesis of a reduced COL1 degradation during fibrosis development, showing that COL1-degradation products collected from the pelvis of the obstructed (fibrotic) kidney were predominantly downregulated compared with non-fibrotic controls (Frattini 2025). Importantly, this downregulation was already observed three days after obstruction, when an increase in collagen protein deposition in the kidney tissue was not yet detectable (Frattini 2025). These findings suggest that impaired collagen degradation may contribute to the early development of kidney fibrosis rather than occurring solely as a consequence of established fibrosis (Frattini 2025). In the current study we investigated the association of COL1 peptides with each fibrotic disease (LDs, CKD and HF) separately and identified peptides that were commonly associated with all three diseases. All of these peptides were fragments of the mature COL1, therefore products of COL1 degradation, and most of them were downregulated in fibrosis, further supporting the concept of reduced collagen degradation during fibrotic remodelling across different tissues.

A plausible explanation for reduced collagen cleavage is the increased cross-linking of collagen fibres in fibrotic tissue. This may be driven by the upregulation of cross-linking enzymes, including lysyl hydroxylase (Piersma and Bank 2019; Remst et al. 2013), lysyl oxidase (Zhang et al. 2020) and transglutaminase (Johnson et al. 2003), and the accumulation of advanced glycation end-products (Koska et al. 2022; Makino et al. 1995; DeGroot et al. 2001) in fibrotic disease. These alterations increase collagen stability and limit its accessibility to MMPs, thereby promoting extracellular matrix accumulation (Panwar et al. 2018; Piersma and Bank 2019; Johnson et al. 2003; DeGroot et al. 2001). Moreover, given the dependence of MMP catalytic activity on zinc, collagen degradation may be further hindered by the consistent downregulation of metallothioneins, resulting in reduced zinc ion availability across fibrotic diseases (Campos 2025). Chronic diseases, including LDs (Gan et al. 2025), CKD (Ying et al. 2023) and HF (Shahim et al. 2023) are major contributors of global morbidity and mortality. However, commonly used clinical tests that assess liver function (LFTs), kidney function (eGFR), and heart injury (troponins or natriuretic peptides) often detect these diseases at late stages, when significant organ damage has occurred, with limited means to stop the progression (Ahmed et al. 2018; Ginès et al. 2022; Rende et al. 2023; Rupprecht et al. 2024; Fang et al. 2026). Therefore, there is a clear clinical need for tools that would enable the early detection of chronic diseases, when lifestyle and pharmacological interventions are more effective at slowing disease progression. Given that fibrosis develops from the early stages of these conditions and progressively contributes to organ dysfunction, its detection may offer an opportunity for earlier therapeutic intervention across chronic diseases (Rupprecht et al. 2024; Pose et al. 2025; Ureche et al. 2020).

Currently the gold standard for the diagnosis of fibrosis is biopsy, an invasive procedure that in some cases leads to health complications and is prone to sampling bias. Therefore, there is a need for the development of methods for the non-invasive assessment of fibrosis. Various imaging and blood/urine-based methods have been developed towards this end. Among imaging techniques, vibration-controlled transient elastography (FibroScan) is the most validated method for liver fibrosis assessment, although confounding factors must be considered to avoid misinterpretation of fibrosis severity (Canivet and Boursier 2023; Castera 2015). For the assessment of cardiac fibrosis, late gadolinium enhancement cardiac magnetic resonance is used in clinical practice, however, it has limited sensitivity for detecting diffuse interstitial fibrosis and requires the administration of gadolinium-based contrast agents (Zhu et al. 2022; Mewton et al. 2011). Although imaging-based methods such as ultrasound, positron emission tomography/computed tomography, and magnetic resonance imaging show potential for the non-invasive assessment of renal fibrosis, their use in clinical practice remains limited (Yuan et al. 2025). Blood-based tests such as aspartate aminotransferase to platelet ratio index and fibrosis-4 index are widely used for liver fibrosis but represent indirect markers influenced by confounders (Canivet and Boursier 2023; Castera 2015). Similarly, circulating biomarkers such as galectin-3 and soluble ST2 have been associated with cardiac fibrosis, yet lack specificity due to elevation in other inflammatory conditions (Zhu et al. 2022). Proposed biomarkers for kidney fibrosis, such as human epididymis protein 4 (HE4) and dickkopf-related protein 3 (DKK3), also reflect broader biological processes beyond fibrosis (Rupprecht et al. 2024). In contrast, biomarkers derived from collagen turnover may provide a more direct assessment of fibrosis. Previous studies have shown that collagen derived peptides not only provide mechanistic insight into the fibrotic process but also could help in the detection and prediction of fibrotic disease. For example, the enhanced liver fibrosis (ELF) test is a widely validated blood test based on the procollagen type III N-terminal propeptide (PIIINP), reflecting collagen type III synthesis, hyaluronic acid and tissue inhibitor of metalloproteinase 1 (Castera 2015). A meta-analysis demonstrated good diagnostic performance of the ELF test in both significant fibrosis (≥ F2) and advanced fibrosis (≥ F3), with summary receiver operating characteristic AUCs of 0.81 and 0.83, respectively (Vali et al. 2020). The levels of PIIINP in urine are also correlated with the degree of kidney fibrosis (Rupprecht et al. 2024). Additionally, the procollagen type I C-terminal propeptide (PICP), reflecting synthesis of COL1, is associated with myocardial fibrosis and can predict adverse outcome in HF patients, while, the cross-linked collagen type I C-terminal telopeptide (CITP), released by the collagenase matrix metalloproteinase-1 and reflecting COL1 degradation, predicts HFpEF incidence (Martin et al. 2025). In addition, urinary peptide models including also collagen fragments have demonstrated ability to detect fibrosis in the liver (Bannaga et al. 2020) and kidney (Catanese et al. 2021).

Building on these observations, the present study identified a common urinary COL1 degradation signature across patients with fibrosis affecting different organs using peptidomics. A model developed based on this signature was able to assess fibrosis in an independent validation cohort, independently of its specific organ localisation. These findings support the concept that fibrosis is driven by shared extracellular matrix remodelling processes across different tissues. Since fibrosis could be interpreted as disturbance in COL1 homeostasis resulting from both an increased COL1 production and a decreased COL1 degradation, a model based solely on degradation products is unlikely to fully capture the complexity of the disease. Therefore, using a combination of the COL1 degradation-based model with markers of COL1 synthesis may be the most beneficial strategy for fibrosis detection.

The study has several limitations. First, because no urine samples from patients with confirmed heart fibrosis were available, samples from patients with HFpEF were investigated based on the assumption that HFpEF is tightly linked to cardiac fibrosis. This approach is supported by a previous study by Hahn et al. (Hahn et al. 2020), who analysed myocardial tissue from a large cohort of HFpEF patients and found that 93% exhibited cardiac fibrosis. Moreover, because most LDs and CKD patients used in the study had already late-stage disease, future validation of the model will be needed to determine its performance in detecting fibrosis at early stages. The low eGFR in the CKD discovery cohort cannot be matched with the controls, as low eGFR defines CKD. Influence of impaired kidney function on the urinary peptide levels in this cohort cannot be formally excluded, however peptide intensities are normalized using 29 internal standard collagen fragments that are generally unaffected by disease. As such it is reasonable to assume that reduced eGFR does not affect the normalised signal intensities. Controls from population-based studies with confirmed absence of fibrosis were also not available, since in clinical practice fibrosis is assessed based on specific clinical indications. Therefore, the possibility that some control individuals had underlying fibrosis despite the absence of clinical symptoms, resulting in misclassification and affecting the classifier’s specificity, cannot be excluded. However, their clinical characteristics suggest that the majority were unlikely to have significant fibrotic disease, supporting their use as comparator groups in the analyses. Additionally, the exact mechanisms underlying the generation of COL1-derived urinary biomarkers are not fully understood. Although in a previous study we modelled the generation of COL1 peptides as the result of protease activity (Mina et al. 2025), the precise tissue origin of the peptides cannot be traced. Accordingly, these biomarkers reflect systemic collagen turnover, which may also be influenced by processes beyond fibrosis. Nevertheless, a previous study identifying liver fibrosis biomarkers showed gradual changes in the abundance of urinary collagen-derived peptides with liver disease severity, suggesting that the observed changes reflect fibrosis in liver (Bannaga et al. 2020). Moreover, the systemic nature of the common fibrosis biomarkers identified in our study may represent an advantage for fibrosis detection, as fibrosis can be identified independently of the organ of origin. In addition, the cross-sectional design and absence of longitudinal follow-up data limit conclusions regarding the causal relationship between altered COL1 degradation and fibrosis development and progression. Another limitation is that comparison with other fibrosis biomarkers could not be performed, as these biomarkers were not measured in the investigated cohorts.

Future studies should validate the model in patients with early-stage fibrosis and in at-risk populations, including individuals with ageing-related risk factors, hypertension, obesity, diabetes and chronic low-grade inflammation. These studies should also compare its performance with established fibrosis markers and investigate whether combining the model with existing markers, particularly markers of collagen synthesis, further improves its performance. Ultimately, prospective clinical trials in at-risk populations could investigate whether individuals with a positive fibrosis signature before clinically apparent organ damage benefit from earlier preventive or therapeutic interventions with potential anti-fibrotic effects, such as sodium-glucose cotransporter 2 (SGLT2) inhibitors, or from closer clinical monitoring. In addition, such a signature could potentially facilitate the development and evaluation of anti-fibrotic therapies by enabling biomarker-based patient stratification according to shared molecular mechanisms rather than clinical symptoms, similar to approaches used in oncology (Tsimberidou et al. 2020).

## Conclusion

By investigating naturally occurring peptides in urine, the study revealed a common COL1 degradation signature across different diseases with fibrotic component, including LDs, CKD and HF. A model based on this signature could detect fibrosis in an independent validation cohort, highlighting the potential of urinary biomarkers as a tool for a non-invasive assessment of fibrosis, irrespective of organ origin. The predominance of downregulated COL1 degradation products in this signature further supports the hypothesis that attenuated COL1 degradation contributes to fibrotic remodelling across different organs. Rather than replacing organ-specific diagnostics, this approach could contribute to the early identification of fibrotic remodelling and support treatment selection before irreversible organ damage occurs.

## Supplementary Information

Additional file 1.

Additional file 2.

## Data Availability

The datasets used and/or analysed during the current study are available from the corresponding author on reasonable request.

## Abbreviations

AUC: Area under the receiver operating characteristic curve
CE-MS: Capillary electrophoresis coupled to mass spectrometry
CI: Confidence interval
CITP: Collagen type I C-terminal telopeptide
CKD: Chronic kidney disease
COL1: Collagen type I
COL1A1: Collagen type I alpha 1 chain
COL1A2: Collagen type I alpha 2 chain
DKK3: Dickkopf-related protein 3
ELF: Enhanced liver fibrosis
eGFR: Estimated glomerular filtration rate
HE4: Human epididymis protein 4
HF: Heart failure
HFpEF: Heart failure with preserved ejection fraction
IFTA: Interstitial fibrosis and tubular atrophy
IQR: Interquartile range
LDs: Liver diseases
LFTs: Liver function tests
MMP: Matrix metalloproteinase
NYHA: New York Heart Association
PICP: Procollagen type I C-terminal propeptide
PIIINP: Procollagen type III N-terminal propeptide
SGLT2: Sodium‐glucose cotransporter 2
SVM: Support vector machine

## References

[CR1] Abdelhameed F, Kite C, Lagojda L, Dallaway A, Chatha KK, Chaggar SS, et al. Non-invasive scores and serum biomarkers for fatty liver in the era of metabolic dysfunction-associated steatotic liver disease (MASLD): a comprehensive review from NAFLD to MAFLD and MASLD. Curr Obes Rep. 2024;13:510–31. 10.1007/s13679-024-00574-z PMC11306269 38809396

[CR2] Ahmed Z, Ahmed U, Walayat S, Ren J, Martin DK, Moole H, et al. Liver function tests in identifying patients with liver disease. Clin Exp Gastroenterol. 2018;11:301–7. 10.2147/CEG.S160537 PMC6112813 30197529

[CR3] An DW, Yu YL, Martens DS, Latosinska A, Zhang ZY, Mischak H et al. Statistical approaches applicable in managing OMICS data: Urinary proteomics as exemplary case. Mass Spectrom Rev. 2023; 1–18. 10.1002/mas.21849 37143314

[CR4] Antar SA, Ashour NA, Marawan ME, Al-Karmalawy AA. Fibrosis: types, effects, markers, mechanisms for disease progression, and its relation with oxidative stress, immunity, and inflammation. Int J Mol Sci. 2023;24:4004. 10.3390/ijms24044004 PMC9963026 36835428

[CR5] Bannaga AS, Metzger J, Kyrou I, Voigtländer T, Book T, Melgarejo J, et al. Discovery, validation and sequencing of urinary peptides for diagnosis of liver fibrosis—A multicentre study. EBioMedicine. 2020;62:103083. 10.1016/j.ebiom.2020.103083 PMC7648178 33160210

[CR6] Benjamini Y, Hochberg Y. Controlling the false discovery rate: a practical and powerful approach to multiple testing. J R Stat Soc Ser B Methodol. 1995;57:289–300.

[CR7] Bülow RD, Boor P. Extracellular matrix in kidney fibrosis: more than just a scaffold. J Histochem Cytochem. 2019;67:643–61. 10.1369/0022155419849388 PMC6713975 31116062

[CR8] Campos MAJ, Stroggilos R, Schanstra J-P, Vlahou A, Brunet M, Jonas J-C et al. Pan-fibrotic gene expression signature in major chronic diseases by integrative bulk and single-cell transcriptomic analyses. medRxiv 2025; 10.1101/2025.10.07.25337472.

[CR9] Canivet CM, Boursier J. Screening for liver fibrosis in the general population: where do we stand in 2022? Diagnostics (Basel). 2023;13:1–15. 10.3390/diagnostics13010091 PMC9818643 36611384

[CR10] Castera L. Noninvasive assessment of liver fibrosis. Dig Dis. 2015;33:498–503. 10.1159/000374097 26159265

[CR11] Catanese L, Siwy J, Mavrogeorgis E, Amann K, Mischak H, Beige J, et al. A novel urinary proteomics classifier for non-invasive evaluation of interstitial fibrosis and tubular atrophy in chronic kidney disease. Proteomes. 2021;9:32. 10.3390/proteomes9030032 PMC8293473 34287333

[CR12] DeGroot J, Verzijl N, Budde M, Bijlsma JWJ, Lafeber FPJG, TeKoppele JM. Accumulation of advanced glycation end products decreases collagen turnover by bovine chondrocytes. Exp Cell Res. 2001;266:303–10. 10.1006/excr.2001.5224 11399058

[CR13] Devos H, Zoidakis J, Roubelakis MG, Latosinska A, Vlahou A. Reviewing the regulators of COL1A1. Int J Mol Sci. 2023. 10.3390/ijms241210004. PMC10298483 37373151

[CR14] Fang X, Xu Y, Zhao Y, Li L, Cheng Z, Zhang Z et al. Beyond Traditional Screening: The Future of Heart Failure Detection With Biomarkers and AI. INew Med. (2026); n/a: e70051.

[CR15] Frantzi M, Keller F, Latosinska A, Beige J, Mebazaa A, Caillard A, et al. Urinary collagen peptides predict mortality. Proteomics. 2026. 10.1002/pmic.70131. PMC13327709 41954080

[CR16] Frattini T, Breuil B, Buléon M, Feuillet G, Chabbert M, Delecroix E et al. Reduced collagen degradation in pelvic urine precedes kidney fibrosis induced by unilateral ureteral obstruction. (2025). 10.21203/rs.3.rs-6872444/v1 PMC12749882 41272289

[CR17] Gan C, Yuan Y, Shen H, Gao J, Kong X, Che Z, et al. Liver diseases: epidemiology, causes, trends and predictions. Signal Transduct Target Ther. 2025;10:33. 10.1038/s41392-024-02072-z PMC11794951 39904973

[CR18] Ginès P, Castera L, Lammert F, Graupera I, Serra-Burriel M, Allen AM, et al. Population screening for liver fibrosis: toward early diagnosis and intervention for chronic liver diseases. Hepatology. 2022;75:219. 10.1002/hep.32163 34537988

[CR19] Good DM, Zürbig P, Argilés À, Bauer HW, Behrens G, Coon JJ, et al. Naturally occurring human urinary peptides for use in diagnosis of chronic kidney disease. Mol Cell Proteomics. 2010;9:2424–37. 10.1074/mcp.M110.001917 PMC2984241 20616184

[CR20] Hahn VS, Yanek LR, Vaishnav J, Ying W, Vaidya D, Lee YZJ, et al. Endomyocardial biopsy characterization of heart failure with preserved ejection fraction and prevalence of cardiac amyloidosis. JACC Heart Fail. 2020;8:712–24. 10.1016/j.jchf.2020.04.007 PMC7604801 32653448

[CR22] He T, Mischak M, Clark AL, Campbell RT, Delles C, Díez J, et al. Urinary peptides in heart failure: a link to molecular pathophysiology. Eur J Heart Fail. 2021;23:1875–87. 10.1002/ejhf.2195 PMC9291452 33881206

[CR23] Ho DE, Imai K, King G, Stuart EA. MatchIt: nonparametric preprocessing for parametric causal inference. J Stat Softw. 2011;42:1–28.

[CR24] Hussain Y, Bannaga A, Fisher N, Krishnamoorthy A, Kimani P, Malik A, et al. The fatty liver, cirrhosis, and liver cancer study (TENDENCY): protocol for a multicenter case-control study. JMIR Res Protoc. 2023;12:1–7. 10.2196/44264 PMC10267778 37256650

[CR25] Jantos-Siwy J, Schiffer E, Brand K, Schumann G, Rossing K, Delles C, et al. Quantitative urinary proteome analysis for biomarker evaluation in chronic kidney disease. J Proteome Res. 2009;8:268–81. 10.1021/pr800401m 19012428

[CR26] Johnson TS, El-Koraie AF, Skill NJ, Baddour NM, El Nahas AM, Njloma M, et al. Tissue transglutaminase and the progression of human renal scarring. J Am Soc Nephrol. 2003;14:2052–62. 10.1097/01.asn.0000079614.63463.dd 12874459

[CR27] Kim KK, Sheppard D, Chapman HA. TGF-β1 signaling and tissue fibrosis. Cold Spring Harb Perspect Biol. 2018;10:1–34. 10.1101/cshperspect.a022293 PMC5880172 28432134

[CR28] Koska J, Gerstein HC, Beisswenger PJ, Reaven PD. Advanced glycation end products predict loss of renal function and high-risk chronic kidney disease in type 2 diabetes. Diabetes Care. 2022;45:684–91. 10.2337/dc21-2196 PMC8918197 35051276

[CR29] Ku JC, Raiten J, Li Y. Understanding fibrosis: mechanisms, clinical implications, current therapies, and prospects for future interventions. Biomed Eng Adv. 2024;7:100118.

[CR30] Latosinska A, Siwy J, Mischak H, Frantzi M. Peptidomics and proteomics based on CE-MS as a robust tool in clinical application: the past, the present, and the future. Electrophoresis. 2019;40:2294–308. 10.1002/elps.201900091 31054149

[CR31] Lee CJM, Kosyakovsky LB, Khan MS, Wu F, Chen G, Hill JA, et al. Cardiovascular, kidney, liver, and metabolic interactions in heart failure: breaking down silos. Circ Res. 2025;136:1170–207. 10.1161/CIRCRESAHA.125.325602 PMC12125648 40403106

[CR32] Makareeva E, Leikin S. Collagen structure, folding and function. In: Shapiro JR, Byers PH, Glorieux FH, Sponselle PD, editors. Osteogenes imperfecta. Acad Press; 2014. p. 71–84.

[CR33] Makino H, Shikata K, Hironaka K, Kushiro M, Yamasaki Y, Sugimoto H, et al. Ultrastructure of nonenzymatically glycated mesangial matrix in diabetic nephropathy. Kidney Int. 1995;48:517–26. 10.1038/ki.1995.322 7564121

[CR34] Martens DS, Thijs L, Latosinska A, Trenson S, Siwy J, Zhang ZY, et al. Urinary peptidomic profiles to address age-related disabilities: a prospective population study. Lancet Healthy Longev. 2021;2:e690–703. 10.1016/S2666-7568(21)00226-9 PMC8566278 34766101

[CR35] Martin EM, Chang J, González A, Genovese F. Circulating collagen type I fragments as specific biomarkers of cardiovascular outcome risk: where are the opportunities? Matrix Biol. 2025;137:19–32. 10.1016/j.matbio.2025.03.001 PMC11986567 40037418

[CR36] Mavrogeorgis E, Mischak H, Latosinska A, Siwy J, Jankowski V, Jankowski J. Reproducibility evaluation of urinary peptide detection using CE-MS. Molecules. 2021;26:7260. 10.3390/molecules26237260 PMC8658976 34885840

[CR37] Mavrogeorgis E, Mischak H, Latosinska A, Vlahou A, Schanstra JP, Siwy J, et al. Collagen-derived peptides in CKD: a link to fibrosis. Toxins (Basel). 2022;14:1–13. 10.3390/toxins14010010 PMC8781252 35050988

[CR38] McKleroy W, Lee TH, Atabai K. Always cleave up your mess: targeting collagen degradation to treat tissue fibrosis. Am J Physiol Lung Cell Mol Physiol. 2013;304:L709–21. 10.1152/ajplung.00418.2012 PMC3680761 23564511

[CR39] Mewton N, Liu CY, Croisille P, Bluemke D, Lima JAC. Assessment of myocardial fibrosis with cardiovascular magnetic resonance. J Am Coll Cardiol. 2011;57:891–903. 10.1016/j.jacc.2010.11.013 PMC3081658 21329834

[CR40] Mina IK, Mavrogeorgis E, Siwy J, Stojanov R, Mischak H, Latosinska A, et al. Multiple urinary peptides display distinct sex-specific distribution. Proteomics. 2024;24:e2300227. 10.1002/pmic.202300227 37750242

[CR41] Mina IK, Iglesias-Martinez LF, Ley M, Fillinger L, Perco P, Siwy J, et al. Investigation of the urinary peptidome to unravel collagen degradation in health and kidney disease. Proteomics. 2025;25:e202400279. 10.1002/pmic.202400279 PMC12205280 39740102

[CR42] Mischak H, Vlahou A, Ioannidis JPA. Technical aspects and inter-laboratory variability in native peptide profiling: the CE-MS experience. Clin Biochem. 2013;46:432–43. 10.1016/j.clinbiochem.2012.09.025 23041249

[CR43] Mohammed SF, Hussain S, Mirzoyev SA, Edwards WD, Maleszewski JJ, Redfield MM. Coronary microvascular rarefaction and myocardial fibrosis in heart failure with preserved ejection fraction. Circulation. 2015;131:550–9. 10.1161/CIRCULATIONAHA.114.009625 PMC4324362 25552356

[CR44] Panwar P, Butler GS, Jamroz A, Azizi P, Overall CM, Brömme D. Aging-associated modifications of collagen affect its degradation by matrix metalloproteinases. Matrix Biol. 2018;65:30–44. 10.1016/j.matbio.2017.06.004 28634008

[CR45] Parichatikanond W, Luangmonkong T, Mangmool S, Kurose H. Therapeutic targets for the treatment of cardiac fibrosis and cancer: focusing on TGF-β signaling. Front Cardiovasc Med. 2020;7:1–19. 10.3389/fcvm.2020.00034 PMC7075814 32211422

[CR46] Piersma B, Bank RA. Collagen cross-linking mediated by lysyl hydroxylase 2: an enzymatic battlefield to combat fibrosis. Essays Biochem. 2019;63:377–87. 10.1042/EBC20180051 31324706

[CR47] Pose E, Piano S, Thiele M, Fabrellas N, Tsochatzis EA, Ginès P. Moving diagnosis of liver fibrosis into the community. J Hepatol. 2025;83:258–70. 10.1016/j.jhep.2025.01.026 39892822

[CR48] Remst DFG, Blaney Davidson EN, Vitters EL, Blom AB, Stoop R, Snabel JM, et al. Osteoarthritis-related fibrosis is associated with both elevated pyridinoline cross-link formation and lysyl hydroxylase 2b expression. Osteoarthritis Cartilage. 2013;21:157–64. 10.1016/j.joca.2012.10.002 23069856

[CR49] Rende U, Guller A, Goldys EM, Pollock C, Saad S. Diagnostic and prognostic biomarkers for tubulointerstitial fibrosis. J Physiol. 2023;601:2801–26. 10.1113/JP284289 37227074

[CR50] Rossing K, Mischak H, Dakna M, Zu P, Novak J, Julian BA et al. Urinary Proteomics in Diabetes and CKD. J Am Soc Nephrol. 2008; 1283–1290. 10.1681/ASN.2007091025 PMC2440301 18448586

[CR51] Rupprecht H, Catanese L, Amann K, Hengel FE, Huber TB, Latosinska A, et al. Assessment and risk prediction of chronic kidney disease and kidney fibrosis using non-invasive biomarkers. Int J Mol Sci. 2024;25:1–18. 10.3390/ijms25073678 PMC11011737 38612488

[CR52] Shahim B, Kapelios CJ, Savarese G, Lund LH. Global public health burden of heart failure: an updated review. Card Fail Rev. 2023;9:e11. 10.15420/cfr.2023.05 PMC10398425 37547123

[CR53] Shan L, Wang F, Zhai D, Meng X, Liu J, Lv X. Matrix metalloproteinases induce extracellular matrix degradation through various pathways to alleviate hepatic fibrosis. Biomed Pharmacother. 2023;161:114472. 10.1016/j.biopha.2023.114472 37002573

[CR54] Smith BH, Campbell A, Linksted P, Fitzpatrick B, Jackson C, Kerr SM, et al. Cohort profile: Generation scotland: Scottish family health study (GS: SFHS). The study, its participants and their potential for genetic research on health and illness. Int J Epidemiol. 2013;42:689–700. 10.1093/ije/dys084 22786799

[CR55] Thompson KJ, Mckillop IH, Schrum LW. Targeting collagen expression in alcoholic liver disease. World J Gastroenterol. 2011;17:2473–81. 10.3748/wjg.v17.i20.2473 PMC3103805 21633652

[CR56] Tofte N, Lindhardt M, Adamova K, Bakker SJL, Beige J, Beulens JWJ, et al. Early detection of diabetic kidney disease by urinary proteomics and subsequent intervention with spironolactone to delay progression (PRIORITY): a prospective observational study and embedded randomised placebo-controlled trial. Lancet Diabetes Endocrinol. 2020;8:301–12. 10.1016/S2213-8587(20)30026-7 32135136

[CR57] Tsimberidou AM, Fountzilas E, Nikanjam M, Kurzrock R. Review of precision cancer medicine: evolution of the treatment paradigm. Cancer Treat Rev. 2020;86:102019. 10.1016/j.ctrv.2020.102019 PMC7272286 32251926

[CR58] Ureche C, Nedelcu A-E, Sascău RA, Stătescu C, Kanbay M, Covic A. Role of collagen turnover biomarkers in the noninvasive assessment of myocardial fibrosis: an update. Biomark Med. 2020;14:1265–75. 10.2217/bmm-2020-0298 33021388

[CR59] Vali Y, Lee J, Boursier J, Spijker R, Löffler J, Verheij J, et al. Enhanced liver fibrosis test for the non-invasive diagnosis of fibrosis in patients with NAFLD: a systematic review and meta-analysis. J Hepatol. 2020;73:252–62. 10.1016/j.jhep.2020.03.036 32275982

[CR60] Verzijl N, DeGroot J, Thorpe SR, Bank RA, Shaw JN, Lyons TJ, et al. Effect of collagen turnover on the accumulation of advanced glycation end products. J Biol Chem. 2000;275:39027–31. 10.1074/jbc.M006700200 10976109

[CR61] Wan S, Wang S, He X, Song C, Wang J. Noninvasive diagnosis of interstitial fibrosis in chronic kidney disease: a systematic review and meta-analysis. Ren Fail. 2024;46:2367021. 10.1080/0886022X.2024.2367021 PMC11216256 38938187

[CR62] Wynn TA, Ramalingam TR. Mechanisms of fibrosis: therapeutic translation for fibrotic disease. Nat Med. 2012;18:1028–40. 10.1038/nm.2807 PMC3405917 22772564

[CR63] Ying M, Shao X, Qin H, Yin P, Lin Y, Wu J, et al. Disease burden and epidemiological trends of chronic kidney disease at the global, regional, national levels from 1990 to 2019. Nephron. 2023;148:113–23. 10.1159/000534071 PMC10860888 37717572

[CR64] Yuan T, Wang H, Kang T, Wu W, Ou S. Advancements in the non-invasive diagnosis of renal fibrosis. Front Med. 2025;12:1646412. 10.3389/fmed.2025.1646412 PMC12343605 40809416

[CR65] Zhang Z, Staessen JA, Thijs L, Gu Y, Liu Y, Jacobs L, et al. Left ventricular diastolic function in relation to the urinary proteome: a proof-of-concept study in a general population. Int J Cardiol. 2014;176:158–65. 10.1016/j.ijcard.2014.07.014 PMC4155932 25065337

[CR66] Zhang XQ, Li X, Zhou WQ, Liu X, Huang JL, Zhang YY, et al. Serum lysyl oxidase is a potential diagnostic biomarker for kidney fibrosis. Am J Nephrol. 2020;51:907–18. 10.1159/000509381 33152735

[CR67] Zhao X, Kwan JYY, Yip K, Liu PP, Liu FF. Targeting metabolic dysregulation for fibrosis therapy. Nat Rev Drug Discov. 2020;19:57–75. 10.1038/s41573-019-0040-5 31548636

[CR68] Zhu L, Wang Y, Zhao S, Lu M. Detection of myocardial fibrosis: where we stand. Front Cardiovasc Med. 2022;9:926378. 10.3389/fcvm.2022.926378 PMC9557071 36247487

